# Support, Inclusion, and Special Education Teachers' Attitudes toward the Education of Students with Autism Spectrum Disorders

**DOI:** 10.1155/2012/259468

**Published:** 2012-02-23

**Authors:** Isabel R. Rodríguez, David Saldaña, F. Javier Moreno

**Affiliations:** Departamento de Psicología Evolutiva y de la Educación, Facultad de Psicología, C/Camilo José Cela, s/n, 41018 Sevilla, Spain

## Abstract

This study is aimed at assessing special education teachers' attitudes toward teaching pupils with autism spectrum disorders (ASDs) and at determining the role of variables associated with a positive attitude towards the children and their education. Sixty-nine special education teachers were interviewed. The interview included two multiple-choice Likert-type questionnaires, one about teachers' attitude, and another about teachers' perceived needs in relation to the specific education of the pupil with ASD. The study shows a positive view of teachers' expectations regarding the education of pupils with ASD. A direct logistic regression analysis was performed testing for experience with the child, school relationship with an ASD network and type of school (mainstream or special) as potential predictors. Although all three variables are useful in predicting special education teachers' attitudes, the most relevant was the relationship with an ASD network. Need for information and social support are the relatively highest needs expressed by teachers.

## 1. Introduction

Positive teacher attitudes are an important predictor of the successful education of children with disabilities, including those with autism spectrum disorders (ASDs) [1, 2]. However, the severity and pervasiveness ASD often leads to the teaching and inclusion of this group of pupils to be seen as especially complex [3]. Even teachers of recognized professional competence often consider themselves less able to deal with these students than with those with any other form of special needs [4–7]. 

Research into teachers' attitudes towards inclusion and students with disabilities has shown that they are very much influenced by variables such as experience, training, and perception of available resources and support [8–13]. Greater experience in inclusive educational contexts favours a more positive attitude toward the education of students with special needs in mainstream classrooms [10, 11]. Giangreco [9], for example, found that in a class where students with severe disabilities were included, teacher attitudes changed over time from initial resistance to a more favourable perception.

Teacher training also has a powerful influence on the development of attitudes toward inclusion, especially when it incorporates related and specific professional abilities [8].

Another aspect that influences the way attitudes are configured is teacher perception of available resources. In a study with 1430 teachers with experience in inclusive settings, three types of resources were deemed necessary: training, support from a team of experts, and support in the classroom [13]. The support of experts and other practitioners is especially valuable when it is accompanied by appropriate collaboration [12].

These dimensions (experience, training, and perception of available resources and support) are also relevant in the case of attitudes towards working with children with ASD. Teacher training, for example, is particularly important here to the specific nature of the needs of these students and prevailing misconceptions surrounding autism. Previous research has shown the necessity of updating and expanding on teachers' knowledge base on autism [4–6, 14–17]. In a related study, Jennett et al. [16] explored professional self-efficacy and burn-out in teachers working with these children. In the survey of 64 teachers applying two different teaching methods, relatively low burn-out and high self-efficacy rates were found. This would support the idea that clear practical and theoretical frameworks are necessary for teachers involved in education of children with autism.

Presence and collaboration of support staff is another relevant factor to be considered. Simpson et al. [3] indicate that teachers are prepared to teach students with ASD if this occurs in the context of collaboration with special education teachers and support staff and with other additional resources. This is a need often mentioned by teachers in surveys about their perception of autism (e.g., [17]) (see [1, 18, 19] for studies on how the specific context of support can determine its effects).

 The present study is part of a larger project aimed at determining the status of provision for special educational needs of pupils with ASD in the city of Seville (Spain) [20–24]. Seville has a population of approximately 700,000 inhabitants, of which 95,861 are children in Kindergarten, compulsory primary and secondary education and special education. A total of 165 students with ASD were registered as part of a wider study in the schools of the city. A total of 37% of these students was diagnosed with autism disorder, 33.3% as PDD-NOS, 8.5% as Asperger's syndrome, and 0.6% as Rett syndrome, with an additional 20.6% cases diagnosed as autism spectrum disorders without additional specification.

The families of these children were also interviewed within this broader study. They were asked about perceived needs in their children's education and their satisfaction with the educational system (see [20, 21] for additional information). In general, families' satisfaction was high, although it was largely dependant on the child's educational placement (in a special or regular school). The variables most related to a positive perception were provision of resources and teacher training.

The study also intended to collect teachers' perceptions and demands in order to plan for future support and training schemes. Most of the teachers involved in the education of these children have a college degree in special education (although not specifically related to autism) and sometimes receive supplementary training in autism. They can teach in special or mainstream schools. In mainstream settings, they will be teaching children with ASD or other disabilities who spend all or part of their time in a support classroom separate from the other children. Fifty-two percent of the students participating in the overall study were in mainstream schools. In the city of Seville, there is an additional variable to be considered that is central to the present study. Various parental and professional ASD groups provide specific support to teachers. In some cases, these are teachers hired in schools directly funded and run by the support group, but in others they are teachers belonging to special or mainstream schools. Twenty-seven percent of the children with ASD at the time of the study were taught in schools linked to these networks. All their teachers received extra training and support related to teaching children with ASD. This network provided examples of successful inclusion, developed additional means for informal support, contributed to the dissemination of information and positive experiences among practitioners, and aimed at reducing the anxiety related to the inclusion of a new student with autism in the classroom. One of the objectives of the present study was to determine the influence of these networks on the special education teachers' attitudes and needs, over and above their initial specialization and training. Special education teachers were the focus of the study due to the fact that they are the most frequently involved in the teaching of children with ASD. More importantly, whereas primary teachers, that do not have a college degree in special education, are only present in mainstream schools and largely do not receive the ASD networks support, special education teachers are present in different contexts, with some of them receiving network support (training and advice from experts in intervention with children with autism) and some of them not.

The specific aims of the study were, therefore,

1. to assess special education teachers' attitudes toward teaching pupils with ASD and their current needs for support,
2. to analyse the role of variables associated with a positive attitude towards the children and their education, specifically educational placement and provision of support within a specialized network.

## 2. Materials and Method

### 2.1. Participants and Setting

Sixty-nine special education teachers (19% men and 81% women) were interviewed. All of them held a bachelor-level college degree in special education. Forty-three of them had the support of an autism network. A total of 58% of special education teachers were in mainstream schools. Average teaching experience was 10.8 years (SD = 10.0). Thirty percent had been working as teachers for less than four years. A total of 42% had never been involved in the education of a pupil with ASD prior to the present year. The remainder had an average of 6.7 years of experience (SD = 7.2) teaching children with ASD. A total of 64.9% had 5 or less years of experience.

### 2.2. Data Collection and Instruments

All the teachers of the 165 registered children with ASD in the city were contacted through families belonging to support groups or through educational administrative databases. Together with 27 primary teachers also interviewed as part of the wider study respondents were responsible for the education of 80% of the children with ASD in the city. They were all interviewed individually in their schools. The interviewer read the items to the teachers, and the teachers provided the responses to the interviewer. The interview included the following two questionnaires (accessible at http://bscw.rediris.es/pub/bscw.cgi/3932122/):

**Teacher Attitude**

This questionnaire assessed teachers' attitudes towards the teaching of children with autism. It included items on different dimensions that the current literature has considered could influence and modulate educational practices with children with disabilities, such as the following.

- Evaluation and perception of pupils' parents. Items here reflected teacher perceptions of parents' relationships, both with each other and with the teacher.
- Expectations of child's chances of improvement. Educational practitioners' vision on the possibilities of their students to learn is an important influence on their work and was thenconsidered an important dimension to be included in the questionnaire. Items related to development and the autism triad, as well as others on the children's possibilities of improving on these dimensions, were included.
- Emotional response to working with children with ASD. The perception of this kind of work as pleasant or unpleasant was collected.

An initial pool of items was submitted to the evaluation of panel of expert practitioners in the field of autism. The final result was a 21-item multiple-choice Likert-type questionnaire (1 for total disagreement to 6 total agreement) including eight items relative to the perception of the students' parents, seven related to expectations and six to professional self-concept and emotional reactions to working with the child with ASD (see Table 1). A hierarchical variable cluster analysis (squared Euclidean distance and intergroup linkage) confirmed this item structure. The questionnaire presented a satisfactory internal consistency (Cronbach's *α* =  .85).

**Needs Questionnaire**

Teachers were asked about their perceived needs in relation to the specific education of the pupil with ASD that they were teaching with a 22-item Likert-type questionnaire (1—*I do not need this at all*—to 6—*this is important and I need it urgently. I need it now, without delay—*), similar to the teacher attitude questionnaire. This was a Spanish adaptation for this study of the family needs scale [25]. In our case, the items were adapted to the Spanish educational system and to existing teacher and teacher support figures. The adaptation was revised by autism expert practitioners and piloted. The adapted teacher version included a *need for information* subscale, relative to educational strategies relevant to ASD, a* need for social support* subscale, which covered the demands for personal support or instrumental networks, the *need of help to explain to others*, which was related to possible difficulties to communicate the pupils' needs to colleagues, parents or other children, and the *need for added resources*, including material, personal support or organizational changes (see Table 4). Overall internal consistency was good (Cronbach's *α* =  .85). Reliability for sections was also adequate, reaching  .92 for information,  .85 for social support,  .91 for explanation to others, and  .87 for added resources.

## 3. Results

Analyses were carried out using the SPSS15 package, and a significance level of  .05 was used in all comparisons.

### 3.1. Teacher Attitude

The overall mean score for the attitude questionnaire was 4.6 (SD = 0.6) (see Table 1 for item descriptive). School- and family-related items were analysed separately. The mean attitude excluding family items, which we shall subsequently refer to as attitude toward teaching children with ASD, was of 4.9 (SD = 0.6). Mean score for attitude towards the family was significantly lower (*M = *4.3, SD = 1.0), *t*(68) = 3.88, *P* < .001. A median split of the sample according to the teachers' attitude toward teaching children with ASD was carried out, in order to distinguish those with the most and least positive attitudes. This resulted in two groups with mean scores of 4.4 (SD = 0.4) and 5.4 (SD = 0.3), *t*(67) = −11.01, *P* < .001. A similar median split was carried out taking into account only the family-related items. The resulting mean scores were 3.6 (SD = 0.8) for the least favourable and 5.1 (SD = 0.4) for the more favourable, *t*(49.89) = −9.67, *P* < .001 (see Table 2 for the distribution of participants into the two groups).

The distribution of special education teachers' attitude towards teaching children with ASD was analysed in terms of possible attitude predictors. A direct logistic regression analysis was performed with attitude group as outcome and three predictors, years of experience with the present child (one, two, or three or more years), membership of an ASD network (included or not), and type of school (special or mainstream). A test of the full model with all three predictors against a constant-only model was statistically reliable, *X* ^2^(3, *N* = 69) = 12.67, *P* = .005. This indicates that all the predictors, as a set, reliably distinguish between teachers with more and least favourable attitude towards teaching children with ASD. However, a closer observation of Table 3 shows that, according to the Wald statistics, only the membership of a support network seems to reliably predict attitude (*z* = 8.54, *P* = .003). The special education teachers' mean attitude was 5.2 (SD = 0.5) amongst those that were part of a specific support network, as opposed to 4.7 (SD = 0.6) for those that were not, *t*(67) = 3.34, *P* = .001, ES = 0.89. The odds ratio for this variable confirms its importance, as it indicates that receiving support from an ASD network increases by 6.6 the chances of being in the more favourable group. On the other hand, the model only including this variable is also reliably different from the constant-only model, *X* ^2^(1, *N* = 69) = 11.68, *P* = .001, with an odds ratio for the ASD network of 7.1 This second model correctly predicts of 88% placement for the least favourable and 49% for the most favourable group.

None of these variables were useful for a model prediction of attitude towards families. A direct logistic regression analysis indicated that the full model was no better predictor than the constant-only model.

### 3.2. Teacher Needs

Mean score for the needs questionnaire was 2.9 (SD = 1.1) (see Table 4). Not all the teachers' needs seemed to be equally urgent. Whereas the mean score for need for information was 3.0 (SD = 1.2), social support was 3.0 (SD = 1.1), need to explain was 2.6 (SD = 1.3) and added resources 2.7 (SD = 1.3). A repeated-measures ANOVA showed the differences among needs to be statistically significant, *F*(3, 204) = 5.87, *P* = .001, *η* ^2^ = 0.079. Pairwise Bonferroni corrected comparisons were significant at the  .05 level between need to explain to others and information and need for social support.

A median split according to need for information was carried out. Average scores were 1.9 (SD = 0.6) for the lower demanding group and 4.0 (SD = 0.6) for the high demand group, *t*(67) = −13.90, *P* < .001, ES = 3.55. A direct logistic regression analysis was performed with information demand group (high or low) as outcome and three predictors, time of contact with present child, relationship with ASD network, and type of school. The comparison of the full model against a constant-only model was once again statistically reliable, *X* ^2^(3, *N* = 69) = 12.03, *P* = .007. This indicates that all the predictors, as a set, reliably distinguish between teachers with pressing and low demands for more information about ASD.  Table 5 shows that, according to the Wald statistics, only the type of school, special or mainstream, seems to reliably predict need of information, *z* = 10.04, *P* = .002. In addition, the odds ratio indicates that the change in condition, from being a teacher in a special to a mainstream school, increases by 6.4 the chances of being in the more demanding group. A model with only the type of school is also reliably different from the constant-only model, *X* ^2^(1, *N* = 69) = 9.15, *P* = .002. The prediction success in this last model was high for the high-information-demanding teachers (75%), but not so high for the low-demanding teachers (60%). Special education teachers' demand score for information was 2.5 (SD = 1.0) in special schools, as opposed to 3.4 (SD = 1.3) in mainstream, *t*(67) = 2.945, *P* = .004, ES = 0.73.

Special education teachers in mainstream and special education schools differed on their previous experience with autistic children in general (42.5% of those in mainstream had it versus 79.3% in special schools), *X* ^2^(1, 69) = 9.35, *P* = .002, and in their seniority in their present teaching post (average of 3.2 years, SD = 2.5, for the mainstream special education teachers versus 10.6, SD = 9.0, for the special schools), *t*(31.22) = −4.26, *P* < .001, ES = 1.21. However, inclusion of these variables in the direct logistic regression model was not statistically different from the previously explained model.

Direct logistic regression analyses were also computed for the other types of needs, but in none of them were any of the models significantly different from the constant-only model.

## 4. Discussion

### 4.1. Teachers' Attitude towards Teaching Pupils with ASD

Overall, the study shows a predominantly positive view of teachers' expectations regarding the education of pupils with ASD, their own ability to influence their development, and their relationships with the families. This result is in line with previous studies that show that teachers involved in the education and inclusion of children with ASD are usually favourably engaged [4, 5, 17].

None of the dimensions considered here predicted family perception. This probably indicates a greater influence of specific family factors or general family-school policy aspects which were not included in this study. However, the results do reflect the influence of certain factors on teachers' attitude and expectations towards teaching children with ASD. One of the most relevant is the availability of a support network.

A direct logistic regression analysis was performed testing for experience with the present child, school relationship with an ASD network, and type of school (mainstream or special) as potential predictors. Although all three variables are useful in predicting special education teachers' attitudes, the most relevant was the relationship with an ASD network. Inclusion in a support network increases by more than six the chances of a teacher being in the sample half with a more positive attitude. However, it would seem that the other variables are playing a certain role. A relatively sparse experience with pupils with ASD, and being in a mainstream school, would, jointly with the nonmembership of an ASD network, indicate a higher-risk status in terms of perception of pupils with ASD. Experience with pupils with ASD has been shown to support more positive attitudes toward the inclusion of children with ASD [9–11, 17]. On the other hand, special education schools, with greater provision of resources and staff in our sample, are associated with a more positive attitude. Previous research has shown the need for this type of support for inclusion of children with special needs [12, 13], and Simpson et al. [3] indicate that teachers show a more positive attitude when there are support staff and other resources involved. It could be that mainstream schools do not have sufficient resources to face the educational needs of these children, and this is negatively affecting teacher attitude.

ASD networks also provide added resources, but the fact that positive attitude is associated to membership of an ASD network, over and above the type of school, seems to indicate that the contribution of these contexts is related to additional factors. A very likely possibility is support in training and general commitment to the teaching of children with ASD. Special schools in the city of Seville have a greater provision of support staff and lower classroom ratios than mainstream schools. However, they are not, in general, ASD specific in their student intake or the training of their staff. Jennett et al. [16] showed that commitment to a general educational framework helped staff in reducing their burn-out rates and increasing self-efficacy. It is possible that the ASD networks in our study, in addition to providing extra or more specific material resources, could be acting in precisely this direction.

### 4.2. The Need for Training and Information

Need of support as expressed by teachers varied in relation to the type of support, with need for information and social support being relatively highest. The social support needs in highest demand are those related to the possibility of meeting experts on ASD. Although included in this section of the questionnaire, it is clearly a more instrumental kind of support, related to the obtaining of information. The recommendations of additional training to improve teachers' knowledge base about autism has been a common conclusion in many previous studies [14, 15, 17]. It is possible that this kind of information demand derives from teacher attitude that teaching children with autism requires a greater degree of specialization.

The direct logistic regression performed on information demand yielded the type of school, mainstream or special, as the main predictor of need for information. Being in a mainstream school seems to increase the chances of a special education teacher being in the high information demanding half of the sample by more than six. It would seem that, independently of the important role of the support networks, mainstream schools are a demanding environment that require constant training and update on the part of teachers to a greater degree than special education contexts.

Finally, the relatively lower demand for extra resources in our study should not lead to an understatement of the importance of additional staff and material support for the inclusion of children with special educational needs. In our sample, teachers within special schools and in schools linked to ASD networks have been found to have the most positive attitudes. It has been argued that the improvement of teacher attitude is contingent upon the provision of appropriate resources [3] and that support staff are essential for the inclusion of children with special needs in the classroom [13]. The lack of this support would render the benefits of inclusion unattainable [26–29]. In view of this literature, the lower demand for these resources in our sample is surprising. One of the reasons may be found in the relatively low level of inclusion. Nearly half the children were in special schools or special pull-out units with low teacher-pupil ratios and a certain amount of support staff. The prevalent increase in detection rates, linked to the increase of pupils with less severe forms of autism and therefore a greater presence in regular classrooms, and a currently more determined inclusion policy in Southern Spain, will most surely test the available resources to the limit in a very near future.

Another potential explanation could be that the relatively low demand for extra resources found in our study is the result of a social desirability bias in teachers' responses. However, this explanation seems unlikely, since respondents were able to indicate other kinds of needs as relevant. Also, with respect to their attitudes to the teaching of children with autism, results coincide with those found in other studies using different methodologies.

### 4.3. Inclusion, Teacher Attitude and Teacher Needs

Overall, the teachers' responses in this study suggest that inclusion of pupils with ASD in mainstream settings is a considerable challenge for those involved, needing specific and extraordinary support. If this support, as illustrated by the efforts of the ASD networks in this study, is not implemented, a need for additional information will appear in the teachers of these pupils. The lack of response to this demand could be fostering a less positive attitude toward the education of the pupils with ASD. Schools related to the ASD networks seem to receive the necessary support in terms of ongoing training and a culture of staff commitment and, therefore, favour more positive attitudes toward pupils with ASD, both in mainstream and special schools. A greater need for information about how to teach these children is, however, present in mainstream settings, with or without ASD network membership, probably due to the fact that inclusion is, in itself, a complex process.

## Figures and Tables

**Table 1:** Mean and standard deviation of items included in the teacher attitude questionnaire (scores for negative items have been inverted) (*N* = 69).

Items	Range	Mean	SD
His/her parents have a positive attitude towards him/her	1–6	4.4	1.2
I would prefer to work with children with other types of disability	1–6	5.4	1.1
With a family like this, teachers' work is useless	1–6	5.1	1.4
I am not the right person to work with children like these	1–6	5.7	0.8
We shall probably be able to improve this child's academic abilities	1–6	4.7	1.1
I find it harder to work with this child than with others with different special needs	1–6	4.4	1.6
The family's activities are positive for this child's development	1–6	4.6	1.4
It is very difficult to collaborate with this family	1–6	4.2	1.4
We shall probably be able to improve the social abilities of this child	3–6	4.6	1.1
Even with extra training, I would find it hard to work with this child	1–6	5.1	1.2
I have had very enjoyable moments working with this child	1–6	4.9	1.2
We shall probably be able to help this child adapt to changes in his/her environment	2–6	4.8	1.0
The family is very attentive to the recommendations they receive from the school	1–6	4.6	1.2
We can probably improve this child's language abilities	1–6	4.3	1.4
The family of this child have helped me to better understand the world of autism	1–6	3.5	1.5
We shall probably be able to improve this child's behavior	1–6	4.9	1.1
The knowledge these parents have about their child's special needs is good	1–6	3.8	1.5
I find it very hard to work with this pupil	1–6	4.8	1.2
I am confident that I shall be able to improve my interaction with this child	1–6	4.8	1.2
The contribution of the family to this child's development is very positive	1–6	4.5	1.2
However much I try, I do not feel I shall achieve any improvement in this child	1–6	5.3	1.1

**Table 2:** Distribution of teachers relative to attitude towards the teaching of pupils with ASD, attitude towards their families, ASD network membership, and type of school.

		Relationship with support network			
		Yes		No	
		Type of school		Type of school	
		Mainstream count	Special count	Mainstream count	Special count
Attitude toward ASD teaching	Least favorable	2	1	12	12
	Most favorable	14	4	12	12

	Total	16	5	24	24

Attitude toward families	Least favorable	8	3	13	10
	Most favorable	8	2	11	14

	Total	16	5	24	24

**Table 3:** Direct logistic regression on attitude towards teaching pupils with ASD.

	B	SE	Wald	df	Sig.	Exp. (B)
Type of school	0.347	0.548	0.401	1	0.527	1.415
Experience with pupil	0.139	0.171	0.660	1	0.417	1.149
ASD network membership	1.885	0.645	8.536	1	0.003	6.587
Constant	−0.985	0.574	2.943	1	0.086	0.374

**Table 4:** Mean and standard deviation of the items of the needs questionnaire (*N* = 69).

Items	Range	Mean	SD
Need for information			
I need information about the child's disability	1–5	2.9	1.3
…About how to teach him/her to relate to others	1–6	3.1	1.4
…About how to teach language abilities	1–6	3.2	1.4
…About how to teach academic skills	1–6	3.0	1.5
…About how to interact and communicate with him/her	1–6	2.6	1.4
…About how to reduce behavioral problems	1–6	3.2	1.8

Need for social support			
I need someone in my school with whom I could talk to about this child	1–6	2.6	1.4
I need opportunities to speak to the parents of this pupil	1–5	2.4	1.3
I need time to talk to other teachers and staff about this child	1–6	2.9	1.6
I would like to speak to experts more regularly	1–6	3.4	1.4
I need to read more documentation about this disability	1–6	3.3	1.3
I would like to talk to teachers with pupils like mine	1–6	3.6	1.4

Help to explain to others			
Help in explaining the needs of this pupil to the other children	1–6	2.3	1.5
Help in explaining the child's progress to the parents	1–5	2.4	1.5
Help in advising the child's parents	1–6	3.1	1.5
Help in explaining this child's needs to other staff	1–6	2.4	1.4

Need for added resources			
I need help for the occasions in which I must leave the classroom	1–6	2.8	1.7
I need someone to provide individual support for the child outside the classroom	1–6	2.7	1.7
I need someone to provide individual support for the child in the classroom	1–6	2.5	1.7
I need more physical space to work with this pupil	1–6	2.4	1.5
I need more curricular materials to work with this pupil	1–6	3.5	1.7
I need greater flexibility in my schedule to be able to attend to this child	1–6	2.6	1.6

**Table 5:** Direct logistic regression on need for information.

	B	SE	Wald	df	Sig.	Exp. (B)
Type of school	1.850	0.584	10.043	1	0.002	6.363
Experience with pupil	0.180	0.182	0.971	1	0.324	1.197
ASD network membership	−0.826	0.599	1.899	1	0.168	0.438
Constant	−1.094	0.600	3.330	1	0.068	0.335
